# Comparative High-Pressure Study on Rare-Earth Entropy Fluorite-Type Oxides

**DOI:** 10.1021/acs.cgd.5c01252

**Published:** 2025-12-01

**Authors:** Pablo Botella, David Vie, Leda Kolarek, Neha Bura, Peijie Zhang, Anna Herlihy, Dominik Daisenberger, Catalin Popescu, Daniel Errandonea

**Affiliations:** † Departamento de Física Aplicada-ICMUV, MALTA Consolider Team, 16781 Universitat de Valencia, Valencia 46100, Spain; ‡ Institut de Ciència dels Materials de la Universitat de València, Apartado de Correos 2085, València E-46071, Spain; § Faculty of Chemical Engineering and Technology, University of Zagreb, Zagreb 10000, Croatia; ∥ Diamond Light Source Ltd., Harwell Science & Innovation Campus, Diamond House, Didcot OX11 0DE, U.K.; # CELLS-ALBA Synchrotron Light Facility, Cerdanyola del Vallés, Barcelona 08290, Spain

## Abstract

We report a comparative high-pressure study of two fluorite-type rare-earth oxides with increasing configurational entropy, (CePr)­O_2__–__δ_ and (CePrLa)­O_2__–__δ_. Synchrotron-based powder X-ray diffraction and Raman spectroscopy were carried out up to 30 and 20 GPa, respectively. Both compounds retain the cubic fluorite structure throughout the pressure range explored, although an anomaly is observed between 9 and 16 GPa, characterized by a compressibility plateau and changes in vibrational modes. This behavior is attributed to local lattice distortions and a progressive bond angle bending rather than abrupt phase transitions. In (CePrLa)­O_2−δ_, the onset of amorphization is observed above 22 GPa, highlighting its reduced structural stability. The bulk modulus of both systems shows a slight decrease after the onset of the anomaly, suggesting subtle lattice softening. Raman spectroscopy reveals suppression of the F_2_g mode intensity with increasing cationic disorder, and under compression, partial reordering is evidenced by an increase in the RE–O mode intensity. Our results highlight the complex interplay between configurational entropy, cation size, and pressure in determining the structural stability and vibrational properties of rare-earth high-entropy oxides and provide insight into the mechanisms governing their resilience and local disorder under extreme conditions.

## Graphical abstract

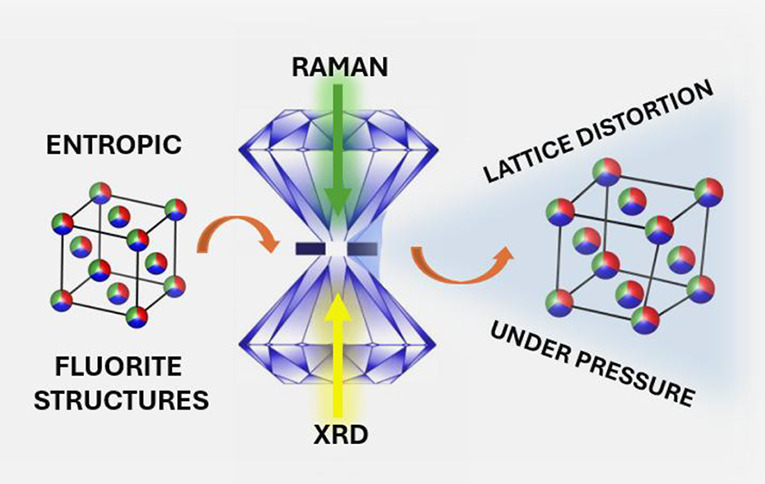

## 1 Introduction

Since their first synthesis in 2015 by Rost et al.,^1^ high-entropy oxides (HEOs) have emerged as a novel class of materials with exceptional structural and functional versatility. These materials, defined by the presence of multiple cationic species in near-equimolar ratios, exhibit remarkable properties such as enhanced thermal stability, mechanical hardness, oxidation resistance, and tunable electrical and magnetic behavior. Rare-earth-based (RE) HEOs,^2^ in particular, are of growing interest due to the rich electronic configurations of *f*-elements, enabling complex interactions and functionalities relevant to energy, electronics, catalysis, and aerospace applications.^3–6^

The structural stabilization of these multicomponent oxides is governed primarily by configurational entropy, which can lower the Gibbs free energy (Δ*G* = Δ*H* – *T*Δ*S*), thereby promoting the formation of single-phase solid solutions that are otherwise immiscible. This entropic contribution is maximized when the constituent elements randomly and in equimolar proportions occupy equivalent lattice positions, as in the fluorite (CaF_2_-type) structure, and becomes increasingly dominant with the number of components (*N*). However, the high degree of chemical disorder can also introduce significant local distortions and raise the internal energy (*U*), making the balance between entropy and enthalpy a delicate and composition-dependent issue.^7,8^

External pressure provides an additional thermodynamic variable to probe the stability of HEOs. Through the PV term in the enthalpy (*H* = *U* + PV), pressure can significantly alter the Gibbs energy landscape, stabilizing or destabilizing phases, modifying local environments, and inducing phase transitions. It can also affect oxygen sublattices, induce amorphization, or even change the valence state of the cations.^7–10^ Hence, high-pressure (HP) studies are crucial to understand the complex interplay between entropy-driven stability and lattice energetics in these systems.

In this work, we present a HP synchrotron XRD and Raman spectroscopy study of (CePr)­O_2__–__δ_ and (CePrLa)­O_2__–__δ_, two fluorite-type rare-earth oxides that represent early steps in a compositional series of increasing configurational entropy. By compressing these compounds up to 30 GPa, we aim to investigate how entropy and cation chemistry influence structural response to pressure, including potential phase transitions or amorphization. These compositions were chosen as intermediate references between CeO_2_ and the high-entropy oxide (CePrLaYSm)­O_2__–__δ_, which has been already studied under pressure, enabling a systematic analysis of the roles played by entropy, oxidation state, and cation size.^7,11–17^ Ce and Pr, both capable of adopting a 4^+^ oxidation state under oxidizing conditions, contribute to fluorite phase stabilization, while the introduction of La^3^^+^ increases entropy and ionic size mismatch. This approach allows us to study the factors governing phase stability and pressure response in complex rare-earth oxide systems.

## 2 Materials and Methods

### 2.1 Synthesis of Materials

Materials used as reagents in the current investigation were Ce­(CH_3_CO_2_)_3_·3H_2_O, Pr­(CH_3_CO_2_)_3_·3H_2_O, and La­(CH_3_CO_2_)_3_·3H_2_O, analytically pure (99.9%) and used as received from Thermo Scientific Chemicals. The starting Ce-, Pr-, or La-containing solutions were prepared by dissolving their respective salts in distilled water. Then, they were combined to obtain Ce–Pr or Ce–Pr–La source solutions having a total cationic concentration of 0.2 M and a total volume of 25 mL. A small amount of acetic acid was added to the solution after the mixture to ensure long-term stability of the solutions. Droplets of these solutions were flash frozen by projection onto liquid nitrogen and then freeze-dried at a pressure of 1–10 Pa and at a temperature of 228 K in a Telstar Cryodos freeze-dryer. In this way, dried solid precursors were obtained as amorphous loose powders. The final oxides were synthesized by thermal decomposition of the amorphous precursor solids. A sample of the selected precursor (ca. 0.2 g) was placed into an alumina boat and introduced into the furnace. The precursor powder was heated under ambient atmosphere at 5 K min^–1^ to a final temperature of 973 K and was held for a period of 1 h. Then, the solid was cooled, leaving the sample inside the furnace.

### 2.2 EDAX Measurements

Rare earth ratios in the solids were determined by energy-dispersive analysis of X-ray (EDAX) on a Thermo Scientific SCIOS 2 scanning electron microscope using an Oxford Ultim Max 170 detector. The operating voltage was 20 kV, and the energy range of the analysis was 0–10 keV.

### 2.3 High-Pressure XRD Experiments

Angle-dispersive X-ray diffraction (ADXRD) experiments were conducted on the (CePr)­O_2__–__δ_ and (CePrLa)­O_2__–__δ_ compounds at the MSPD-BL04 beamline of the ALBA synchrotron. A monochromatic X-ray beam with a wavelength of 0.4246 Å was focused to a 15 μm × 15 μm spot size (full width at half-maximum) using Kirkpatrick–Baez mirrors. The diffraction patterns were recorded using a Rayonix SX165 CCD detector placed at a fixed sample-to-detector distance of 260 mm.^18^

High-pressure conditions were achieved using a membrane-type Almax diamond anvil cell (DAC) equipped with 500 μm culet diamonds. The pressure chamber was prepared by preindenting a stainless-steel gasket and drilling a central hole of approximately 200 μm in diameter and 45 μm in height using an electrical discharge machine. Copper (Cu) powder was loaded alongside the samples and used as an internal pressure calibrant.^19^ A 4:1 methanol–ethanol (ME) mixture was employed as the pressure-transmitting medium (PTM) to ensure quasi-hydrostatic conditions throughout the compression cycle.^20^

Each sample was compressed in steps of approximately 0.4 GPa up to a maximum pressure of 30 GPa, with diffraction patterns collected at each step. Measurements were also performed during decompression to evaluate reversibility and possible hysteresis effects. The collected 2D diffraction images were azimuthally integrated using the Dioptas software package^21^ to obtain conventional one-dimensional diffractograms. Structural analysis of the integrated data was performed using the GSAS-II suite,^22^ while structural visualizations were generated using VESTA software.^23^

### 2.4 High-Pressure Raman Experiments

Raman measurements were performed using a Horiba LabRam HR800 spectrometer equipped with 1200 and 2400 lines/mm gratings located at Diamond light source, at beamline I15. In this study, the 1200 grating was used in combination with a 20× objective. The system includes confocal spatial filtering, and with an entrance slit set to 100 μm, this configuration results in an effective sampling spot of approximately 5 μm. Excitation was provided by a 523 nm (green) laser with a nominal maximum output power of 500 mW. The power was set to 80% of total power. The total time for each point measurement was 10 min. Precautions were taken to minimize possible laser-induced heating effects during Raman measurements. High-pressure conditions were achieved using a LeToullec-style membrane-driven diamond anvil cell (DAC) with a 4-pin configuration. The anvils employed 400 μm culets and were of Boehler-Almax type, made from synthetic type IIa diamonds. The anvils were mounted on tungsten carbide (WC) seats. A ruby chips-sphere along with the sample was introduced to monitor the pressure.^24^ The PTM used was 4:1 ME for a better comparison between XRD and Raman measurements.

## 3 Results and Discussion

### 3.1 Ambient Conditions Structural Characterization

The structural integrity and phase purity of the synthesized fluorite-type compounds, (CePr)­O_2__–__δ_ and (CePrLa)­O_2__–__δ_, were first evaluated through Rietveld refinement of their powder XRD patterns, as shown in Figure 1. The good agreement between the experimental data and the calculated profiles confirms the successful formation of single-phase fluorite structures, assigned to the cubic space group *Fm*3̅*m* (No. 225), in both compositions.^2^ The sharp, symmetric Bragg reflections and minimal residuals in the difference curves further support the high crystallinity and structural homogeneity of the samples.

**Figure 1:**
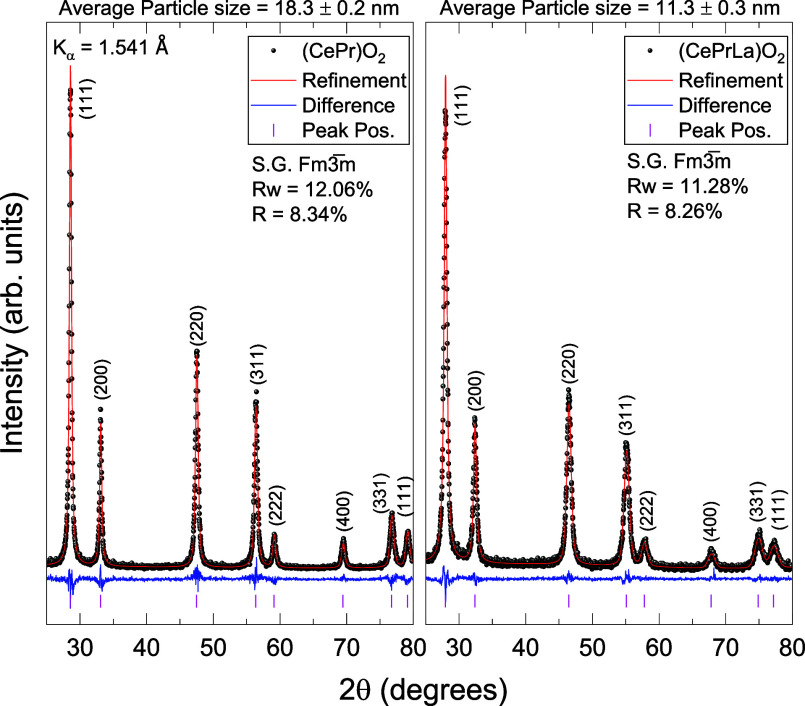
Rietveld refinement of the powder XRD pattern of the as-synthesized compounds. The experimental data are shown as black dots, the fitted pattern is indicated by the red line, and the difference curve (experimental minus calculated) is displayed in blue. The vertical purple ticks represent the expected Bragg peak positions for the fluorite-type structure. Average particle size has been estimated by the Scherrer equation.

Crystallite sizes were estimated using the Scherrer equation, considering up to eight distinct reflections in each diffraction pattern. The calculated average crystallite sizes were 18.3 ± 0.2 nm for (CePr)­O_2__–__δ_ and 11.3 ± 0.2 nm for (CePrLa)­O_2__–__δ_, indicating nanoscale grain dimensions consistent with the synthesis approach.

To verify the homogeneity and nominal composition of the as-synthesized (CePr)­O_2__–__δ_ and (CePrLa)­O_2__–__δ_ powders, EDAX measurements were performed. For each sample, five independent measurements were conducted at different regions of the powder to account for possible local compositional variations. The average atomic percentages obtained from these measurements are summarized in Table 1. The results confirm that the elemental ratios are consistent with the intended stoichiometry (equimolar distribution) to maximize the entropy level, supporting the successful synthesis of homogeneous multicomponent rare-earth oxide phases. Furthermore, the configurational mixing entropy was estimated Table 1, and presented in Figure 2a, together with the values for CeO_2_ and (CePrLaYSm)­O_2__–__δ_, illustrating the low- and medium-entropy character of the studied compounds. The configurational mixing entropy (Δ*S*_m__i__*x*_) was calculated using the standard expression for ideal mixing of *N* cationic species:

$$\Delta S_{\mathrm{mix}}=-R\sum_{i=1}^{N}x_{i}\ln x_{i}$$

where *R* is the universal gas constant and *x_i_* is the molar fraction of each cationic species. For equimolar binary (CePr)­O_2__–__δ_, this yields Δ*S*_m__i__*x*_ = 0.69*R*, and for ternary (CePrLa)­O_2__–__δ_, Δ*S*_m__i__*x*_ = 1.09*R*. We have used the ideal values for the molar fraction 0.5 for (CePr)­O_2__–__δ_, and 0.33 for (CePrLa)­O_2__–__δ_ supported by the measured values by EDAX which are quite close to those.

**Figure 2:**
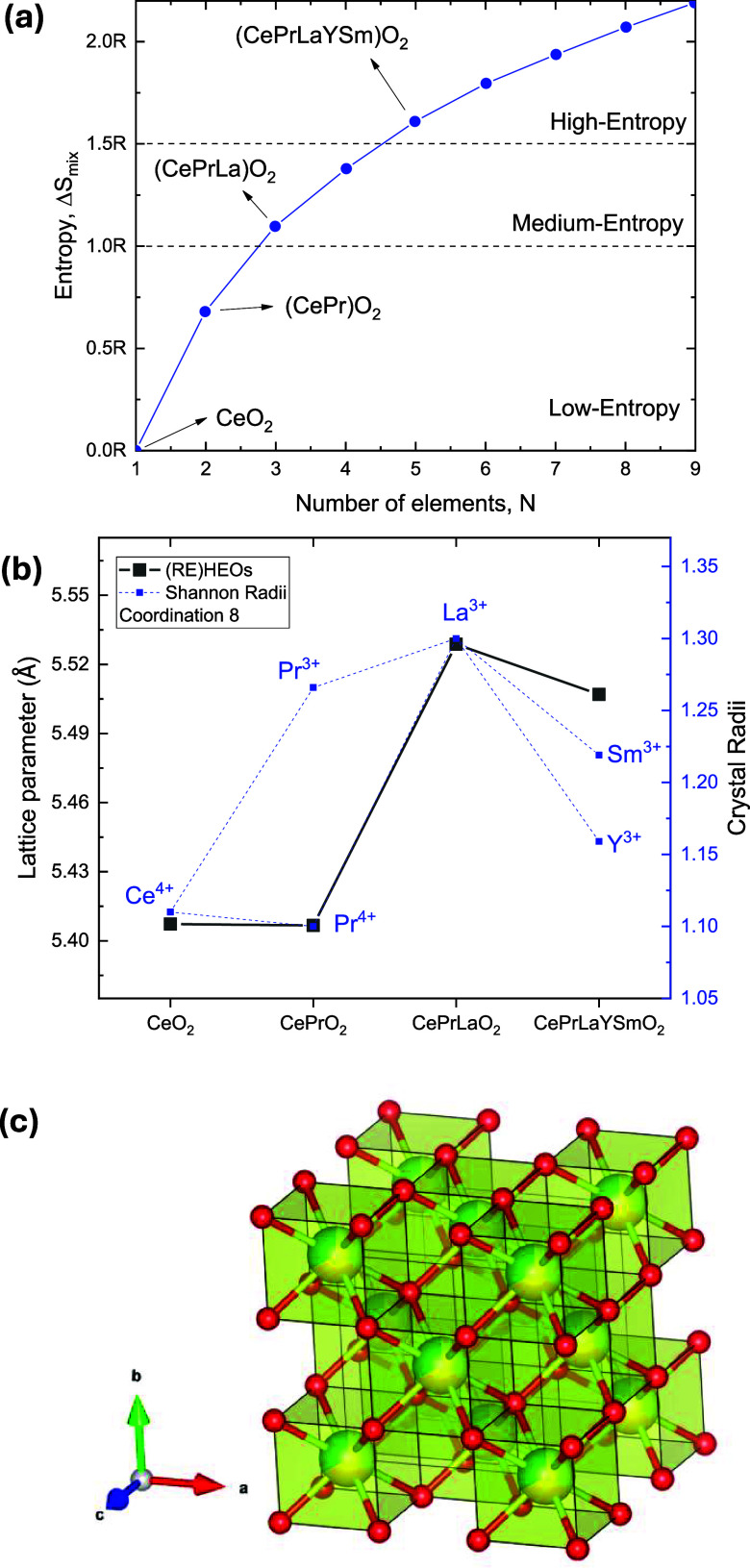
(a) Relationship between entropy value, the number of elements, and the distribution of low, medium, and high entropy ceramics.^25^ (b) Comparative lattice parameter versus ionic radii of the constituent elements. Error bars are within the symbol size. (c) Polyhedral crystal structure representation of the (CePrLa)­O_2_. Oxygen atoms are represented by red atoms. Partially colored atoms are equimolarly represented by Ce, Pr, or La.

**Table 1:** EDAX Results of the As-Synthesized Samples^a^

	theoretical	Ce	Pr	La	Δ*S*_mix_
(CePr)O_2__–d_	50	49.5 ± 0.3	50.5 ± 0.3		0.69*R*
(CePrLa)O_2__–d_	33	32.1 ± 0.3	31.9 ± 0.3	35.97 ± 0.3	1.09*R*

a Values represent the average of five measurements at distinct points on the sample surface.

To further explore the structural implications of increasing configurational entropy, Figure 2b presents a comparative analysis of the lattice parameters and the effective crystal radius of the rare-earth cationic sublattice. The data set includes reference values for binary CeO_2_, as well as for the higher-entropy compound (CePrLaYSm)­O_2__–__δ_, thereby spanning a wide entropy gradient from low to very high configurational complexity.

The observed trend reveals a systematic expansion of the fluorite lattice due to the progressive substitution of smaller tetravalent cations (e.g., Ce^4^^+^) with larger trivalent ions such as Pr^3^^+^, La^3^^+^, Y^3^^+^, and Sm^3^^+^. This behavior aligns with expectations based on the Shannon crystal radii for 8-fold coordination, confirming the sensitivity of the fluorite-type structure to variations in cationic size and composition. In particular, the incorporation of La^3^^+^ leads to a lattice expansion from 5.416(3) Å (CeO_2_) to 5.523(5) Å in (CePrLa)­O_2__–__δ_. For (CePr)­O_2__–__δ_, the lattice parameters suggest the possible presence of Pr^4^^+^ ions, despite the precursor adopting the hexagonal Pr_2_O_3_ structure. A mixed-valence state of Pr^3^^+^/Pr^4^^+^ has previously been reported during the synthesis of (CePrLaYSm)­O_2__–__δ_,^2^ and is believed to contribute, alongside trivalent substitutions, to the formation of oxygen vacancies that help maintain charge neutrality in the system. Although direct spectroscopic techniques (e.g., XPS or XANES) were not employed in this study, the presence of Pr^4^^+^ is inferred based on charge balance considerations and by analogy with similar fluorite-type rare-earth oxides.^2,7^ In these systems, oxygen vacancies and mixed-valence states often coexist, with redox-active elements like Pr facilitating local charge compensation. Under compression, the observed enhancement of the RE–O Raman mode and the concurrent evolution of the VO band (see further in the text) may reflect pressure-induced redistribution of vacancies and subtle redox changes. Nevertheless, these hypotheses require confirmation by future studies involving direct spectroscopic or computational approaches.

Figure 2c presents a structural visualization of the (CePrLa)­O_2__–__δ_ compound, illustrating its face-centered cubic structure, which belongs to the *Fm*3̅*m* space group (No. 225). The CaF_2_-type structure of the rare earth oxides consists of REO_8_ hexahedra (cubes) with a cubic close-packed arrangement, in which each RE atom is surrounded by eight O atoms forming eight RE–O bonds. All the REO_8_ cubes connect with each other by sharing one edge.^26^ The model highlights the random distribution of Ce, Pr, and La atoms within the cationic sublattice, a hallmark of configurational disorder. Oxygen atoms, depicted in red, occupy the fluorite-type coordination sites at the Wyckoff position (1/4, 1/4, 1/4), while the rare-earth cations share the Wyckoff position (0, 0, 0), reflecting a mixed occupancy that underpins the entropic stabilization. This structural model evidence the formation of a homogeneous solid solution, where configurational entropy plays a crucial role in maintaining phase stability despite the ionic size mismatch and charge differences among the constituent cations.

The differences observed in the lattice parameters of the studied compounds at low- and intermediate-entropy compositions further suggest the presence of local lattice distortions, strain fields, and potentially defect formation resulting from increased chemical disorder. Such deviations are typical of high-entropy oxide systems, where the complex cationic environment disrupts ideal lattice packing and generates a distribution of local bonding environments.^1,2^ These findings emphasize that entropy-driven stabilization is intrinsically linked to atomic-scale structural frustration, which may have important implications under high-pressure conditions.

### 3.2 Structural Characterization under High-Pressure Conditions

Selected XRD patterns of (CePr)­O_2__–__δ_ and (CePrLa)­O_2__–__δ_ measured under high-pressure (room-temperature) conditions are shown in Figure 3a,b. The maximum pressure reached in the experiments was about 30 GPa. Qualitatively, it can be observed that both samples retain the cubic fluorite structure throughout the entire pressure range explored. Notably, the pressure has a more pronounced effect on (CePrLa)­O_2__–__δ_, due to the higher lattice distortion introduced by the incorporation of La^3^^+^ cations. This is evidenced by the broader peaks and reduced reflection intensities when compared to (CePr)­O_2__–__δ_ (note that the reflection (111) of all patterns is normalized to 1 and vertically offset for better comparison). Three distinct regions can be identified based on peak evolution with pressure, with region II showing the least variation, as will be further discussed in the text. The recovered patterns also exhibit the fluorite structure with similar features to the starting materials, highlighting the high structural stability of these compounds, likely due to entropy-driven effects.

**Figure 3:**
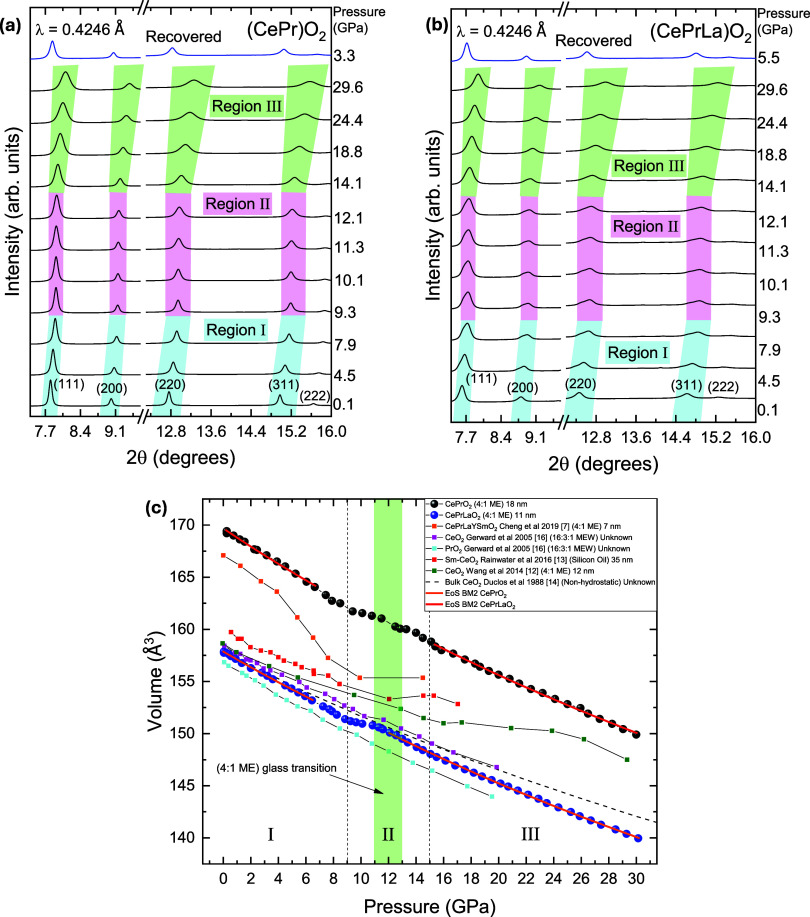
(a) Representative X-ray patterns (λ = 0.4246 Å) of CePrO_2_ and (b) of CePrLaO_2_ at ambient temperature on compression. (c) Unit-cell volume evolution under pressure of both compounds compared to relevant data from literature. The dashed line represents the 2nd order BM EoS fit.

The studied compositions are dominated by CeO_2_, which plays a key role in stabilizing the single-phase in high-entropy oxide structure and provides a CaF_2_-type host lattice for the other rare-earth cations.^2^ Therefore, it is reasonable to expect that their high-pressure behavior will resemble that of pure CeO_2_.^7^ Nevertheless, no signs of the structural phase transition typically reported for CeO_2_ around 30–35 GPa were detected in our experiments.^11–17^ This absence can be explained by considering the limitation of the pressure range studied, the nature of the PTM and the intrinsic configurational entropy of our low- and medium-entropy oxide systems, assuming comparable particle sizes to those in previous studies. In our case, the use of a 4:1 ME mixture as PTM, which is known to become nonhydrostatic above ∼12 GPa,^20^ might have promoted the onset of a phase transition if the structure were inherently unstable, as nonhydrostatic stress conditions tend to reduce the transition pressure.^27^

However, the continued stability of the fluorite structure in (CePr)­O_2−δ_ and (CePrLa)­O_2−δ_ suggests that the configurational entropy introduced by cationic disorder enhances structural resilience against pressure-induced transitions. By contrast, in the higher-entropy oxide (CePrLaYSm)­O_2−δ_,^7^ a loss of long-range order (amorphization) is observed around 16 GPa, indicating that beyond a certain level of chemical complexity, the stabilizing entropy contribution becomes less effective due to excessive disorder. Based on this, one might expect our samples, being of lower entropy, to undergo a transition at a pressure between ∼16 GPa and the 30–35 GPa typical of pure CeO_2_.^11,17^ However, the observed behavior suggests a shift to even higher pressures, or at least stability comparable to CeO_2_.

The absence of a clear phase transition up to 30 GPa reflects a balance between entropy stabilization and the intrinsic resistance of the fluorite framework to deformation. In the case of (CePrLa)­O_2−δ_, however, a broad background contribution becomes apparent above ∼22 GPa, particularly around the first two Bragg reflections (see Figure 4), indicating the onset of partial amorphization. This feature grows with pressure and suggests a progressive collapse of intermediate-range order within the fluorite lattice. Upon decompression, the broad contribution disappears, confirming that the amorphization is reversible. This behavior points to a pressure-driven structural frustration that does not involve irreversible breakdown, but rather a dynamic reorganization of the lattice under stress. Contrary to the observation in (CePrLaYSm)­O_2−δ_, where amorphization was complete at 16 GPa and irreversible at ambient conditions,^7^ the amorphization observed in (CePrLa)­O_2−δ_ is only partial and fully reversible upon decompression.

**Figure 4:**
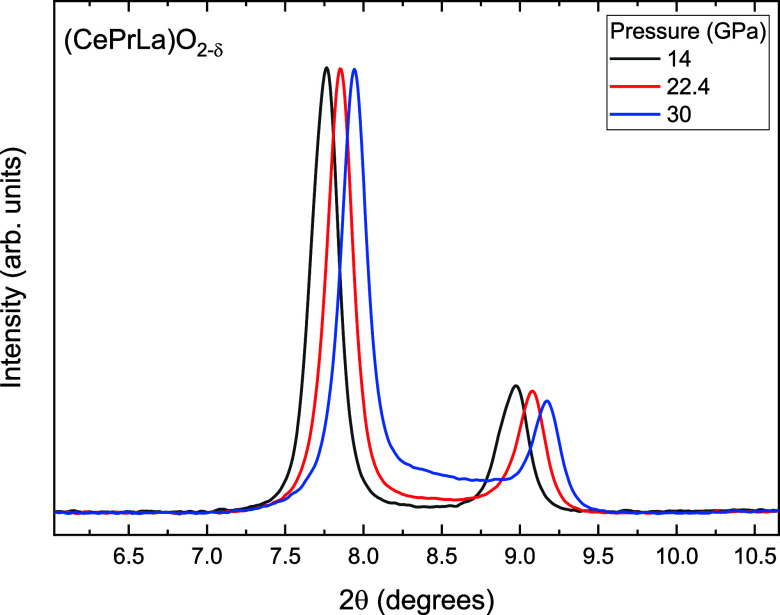
XRD patterns at selected pressure showing the partial amorphous contribution.

It is worth noting that pushing the experiments beyond this pressure range was considered too risky for the instrumentation employed in this study. Before concluding this part of the discussion we would like to note that the stability of (CePr)­O_2__–__δ_ and (CePrLa)­O_2__–__δ_ contrast with behavior of ordered CeO_2_ based alloys like (CeZr)­O_2_ which adopts a cubic pyrochlore structure and undergoes a phase transition at 5 GPa.^28^ Pyrochlore can be described as an ordered derivative of the fluorite structure. In pyrochlore (CeZr)­O_2_, in contrast with fluorite oxides, pressure promotes the formation of oxygen vacancies which favors the observed transition.^29^

The pressure-dependent structural evolution of the synthesized compounds was investigated through volume–pressure (*V*–*P*) measurements, as shown in Figure 3c. The compression behavior of (CePr)­O_2__–__δ_ and (CePrLa)­O_2__–__δ_ is compared with that of related rare-earth oxides reported in the literature.^7,12–14,16^ Both synthesized compounds exhibit a smooth and continuous volume reduction up to approximately 30 GPa (region I and III on Figure 3c), indicating the absence of any abrupt structural phase transitions within this pressure range. However, a noticeable deviation from linear compression behavior is observed between 9 and 16 GPa (region II in Figure 3c), where a plateau and nonuniform volume decrease suggests the presence of local lattice distortions.^7^ This anomaly may correspond to a subtle change in the first derivative of the volume–pressure curve, potentially indicative of a second-order isostructural transition or a gradual evolution in compressibility. Nevertheless, the preservation of the fluorite-type cubic symmetry, the absence of symmetry breaking or new Bragg reflections, and the reversibility observed upon decompression all support an interpretation based on continuous internal rearrangements rather than a distinct thermodynamic phase transition. The green-shaded region in the plot marks the pressure range (∼11–13 GPa) associated with the glass transition of the 4:1 ME PTM.^20^ While deviations from ideal hydrostatic conditions in this range may influence compressibility, the onset of the anomaly at lower pressures (∼9 GPa) suggests that it is primarily intrinsic and not triggered by the nonhydrostatic behavior of the PTM beyond 12 GPa.

Notably, the compressibility of (CePrLa)­O_2__–__δ_ is slightly higher than that of (CePr)­O_2__–__δ_, consistent with its larger initial unit cell volume and the presence of the larger La^3^^+^ cation. This trend aligns with the expected behavior based on ionic size and supports the notion that increasing configurational entropy through cation substitution can subtly modulate the mechanical response of the fluorite lattice.

Comparison with literature data for CeO_2_,^16^ PrO_2_,^16^ and bulk CeO_2_ ^14^ reveals that the synthesized (CePr)­O_2__–__δ_ compound follows a similar compression trend to both CeO_2_ and PrO_2_. However, unlike the findings reported by Gerward et al.,^16^ no anomalies were observed in their study. The anomalies detected in our samples appear to be closely related to the crystalline size of the particles, particularly when they are in the nanometer range, as also noted in the works of Wang^12^ and Rainwater.^13^ These studies report behaviors that resemble our observations, including a slight volume expansion under pressure in Sm-doped CeO_2_ and pure CeO_2_ when silicon oil is used as PTM. However, in those cases, the anomalies typically emerge after the PTM undergoes a glass transition, which differs from our findings.

Rainwater et al.,^13^ partially attributed these effects to the high nonhydrostaticity of the silicone oil used as PTM. In contrast, Wang et al.,^12^ proposed a dual-structure model to explain the anomalous volume expansion observed in nanocrystalline CeO_2_ under pressure. According to their model, the material consists of a rigid amorphous shell surrounding a relatively soft crystalline core. This configuration can lead to an apparent negative compressibility when subjected to nonhydrostatic conditions, particularly with silicone oil as the PTM. Notably, such behavior was not observed when more hydrostatic PTMs, such as ME mixtures, were used. However, a pressure-induced plateau is observed even when ME is employed as the PTM, including in the high-entropy compound (CePrLaYSm)­O_2__–__δ_,^7^ which exhibits a plateau beginning at approximately 9 GPa and transitions into an amorphous phase beyond 16 GPa. According to Cheng et al.,^7^ this pressure-induced plateau arises from a progressive structural disorder that begins around 9 GPa, as revealed by in situ synchrotron X-ray diffraction studies. Instead of further compressing the lattice through bond shortening, the structure accommodates pressure mainly by bending the interatomic bond angles within and between the REO_8_ polyhedral units. While the rigid RE–O bonds remain nearly constant, the continuous distortion of bond angles reduces the long-range crystallographic coherence. As a result, the diffraction peaks cease shifting and gradually broaden and weaken, indicating the onset of topological disorder. This process culminates in the collapse of the long-range order and the formation of an amorphous phase above ∼16 GPa. In addition, the studied compound (CePrLa)­O_2__–__δ_ shows a broader pressure range affected by structural anomalies, spanning from 9 to 16 GPa, as illustrated in Figure 3c, region II. This compound exhibits a larger unit cell volume compared to (CePrLaYSm)­O_2__–__δ_, which can be attributed to the higher concentration of La^3^^+^ cations incorporated into the lattice. These findings underscore the critical role of both particle size and PTM characteristics in determining the high-pressure behavior of rare-earth oxides like (CePr)­O_2__–__δ_ and (CePrLa)­O_2__–__δ_. Nevertheless, further complementary measurements are necessary to fully elucidate the mechanisms behind these anomalies.

To determine the bulk modulus of the studied compounds, the pressure–volume (*P*–*V*) data were fitted using a second-order Birch–Murnaghan (BM) equation of state (EoS). Data points corresponding to regions with structural anomalies were excluded from the fitting. As a result, two sets of bulk modulus values were obtained for each compound: one for the low-pressure region (region I) and one after the structural anomaly.

The bulk moduli for region I (pressure points from 0 to 6 GPa) are shown in Figure 5, alongside those of the precursor compounds for comparison.^12,14,16,30^ The refined EoS parameters for (CePr)­O_2__–__δ_ are *V*_0_ = 157.87(2) Å^3^, *B*_0_ = 205.1(19) GPa, with fixed *B*_0_′ = 4, and a calculated second derivative *B*_0_″ = −0.019 GPa^–1^. For (CePrLa)­O_2__–__δ_, the values are *V*_0_ = 169.68(6) Å^3^, *B*_0_ = 190(4) GPa, *B*_0_′ = 4, *B*_0_″ = −0.0204 GPa^–1^. As expected, the bulk modulus of (CePr)­O_2__–__δ_ lies between that of CeO_2_ (its host lattice) and Pr-containing oxides, reflecting the intermediate nature of its composition. In contrast, (CePrLa)­O_2__–__δ_ exhibits a lower bulk modulus, shifting closer to the values typically found in La-containing oxides, although still within the range of praseodymium oxides. This reduction is attributed to the presence of the additional (third) cation, which distributes the lattice strain among more species and thus reduces the individual influence of each rare-earth element on the overall stiffness of the structure. After the onset of structural anomalies (pressure points from 12 to 30 GPa for (CePr)­O_2__–__δ_ and from 15 to 30 GPa for (CePrLa)­O_2__–__δ_), the bulk moduli of (CePr)­O_2__–__δ_ and (CePrLa)­O_2__–__δ_ decrease slightly to 182.3(11) and 181(3) GPa, respectively. This suggests that structural modification results in a slight enhancement of the compressibility of the lattice.

**Figure 5:**
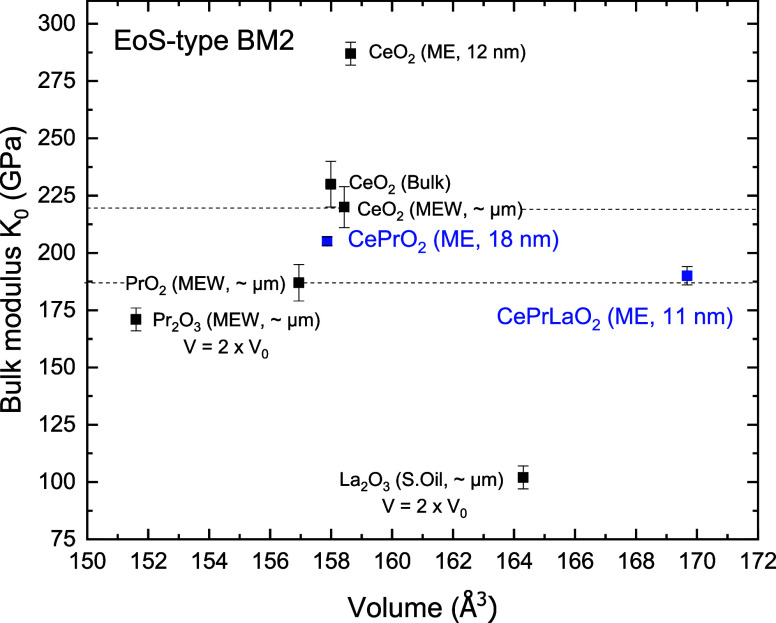
Bulk modulus of the CePrO_2_ and CePrLaO_2_ compounds using different PTMs as indicated compared to their precursor’s bulk modulus.^12,14,16,30^

### 3.3 High-Pressure Conditions Vibrational Characterization

In order to gain deeper insight into the structural characteristics of the studied compounds, Raman spectroscopy was performed at ambient conditions and under high pressure at room temperature. The ambient-condition Raman spectra are shown in Figure 6. Two broad features are observed: the first corresponds to the F_2_g symmetric vibration mode of the 8-fold coordinated RE–O bond, typically located around ∼450 cm^–1^, while the second, at ∼600 cm^–1^, is attributed to the oxygen vacancy (V_O_) band commonly found in high-entropy oxides (HEOs).^7^ In the pure CeO_2_ system, the F_2_g mode is the most intense feature, but its intensity progressively decreases as more cations are introduced into the lattice. This trend is evident when comparing (CePr)­O_2__–__δ_ and (CePrLa)­O_2__–__δ_ to the more chemically disordered (CePrLaYSm)­O_2__–__δ_, where the F_2_g peak is almost completely suppressed. The weakening of the F_2_g mode is attributed to the increasing degree of local symmetry breaking and severe lattice distortion, resulting from the high chemical disorder inherent to multicomponent systems.

**Figure 6:**
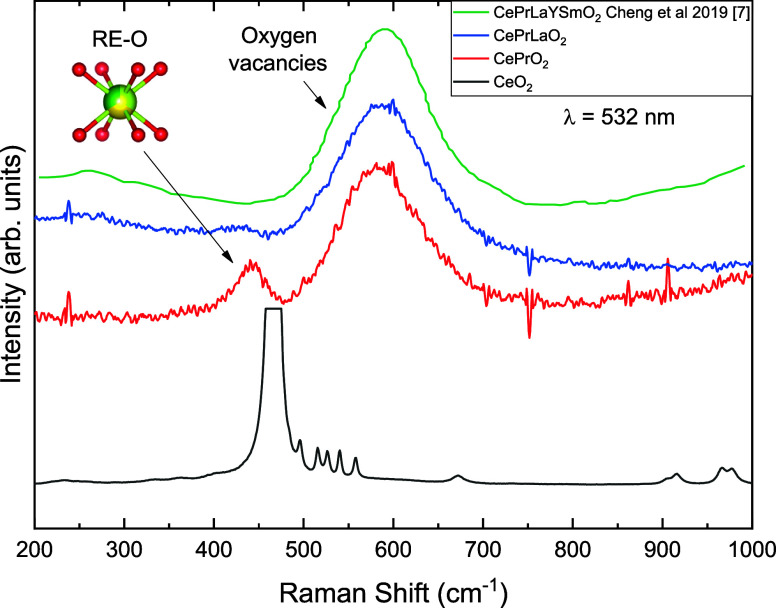
Ambient conditions Raman spectra of the CePrO_2_ and CePrLaO_2_ along with CeO_2_ and CePrLaYSmO_2_ compounds.

Figure 7a shows the representative Raman spectra of (CePr)­O_2__–__δ_ during compression, showing the evolution of the RE–O vibrational mode (∼450 cm^–1^) and the oxygen vacancy (V_O_) band (∼600 cm^–1^). A gradual blue-shift and broadening of both peaks are observed with increasing pressure, along with intensity changes. In the case of (CePrLa)­O_2__–__δ_ (Figure 7b), the Raman spectra show similar trends to those of (CePr)­O_2__–__δ_, although the intensity of the RE–O mode is generally lower. This reduction is consistent with an increased degree of lattice distortion and higher cationic disorder arising from the presence of La^3^^+^ in the structure. The V_O_ band undergoes several notable changes during compression, including variations in intensity and peak shape. As observed in Figure 6a, two distinct peaks are present at ambient pressure, but they progressively merge into a single broad band under increasing pressure. This behavior suggests that the oxygen vacancies evolve under compression, potentially altering their local environment, distribution, and concentration. This behavior may be associated with the relatively high compressibility of the vacancy sites, as well as pressure-induced changes in the oxidation state of praseodymium, particularly the conversion from Pr^3^^+^ to Pr^4^^+^. These factors contribute to the evolving local environment and increased lattice distortion during compression.^7^

**Figure 7:**
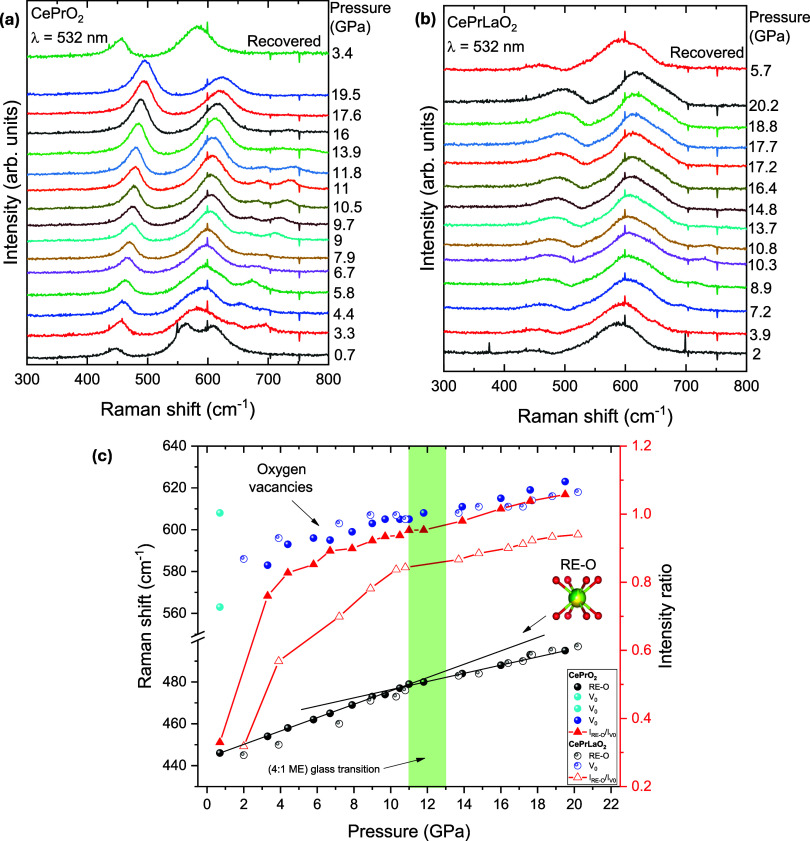
(a) Representative Raman spectra (λ = 532 nm) of CePrO_2_ and (b) CePrLaO_2_ at ambient temperature on compression. (c) Pressure dependence of the Raman shift for the two main features related to RE–O vibrations and oxygen vacancies. The evolution of their intensity ratio (*I*_RE–O_/*I*_V0_) with pressure is also shown. A straight line has been added to the RE–O mode data to aid visualization of the change in slope.

Figure 7c shows the pressure evolution of the Raman modes under pressure as well as the intensity ratio (*I*_RE–O_/*I*_vo_). As the spectra exhibit very broad bands, the peak positions were estimated by tracking the intensity maxima to qualitatively follow their evolution under pressure. The V_O_ band exhibits a linear shift under compression with no notable variation. In contrast, the RE–O band displays a clear change in slope around 10–11 GPa, which correlates with the onset of bond distortion observed in the XRD data. Interestingly, both compounds exhibit an enhanced RE–O mode intensity upon compression, eventually surpassing the intensity of the V_O_ band. This behavior may indicate a pressure-induced rearrangement or ordering in the local environment, where the RE–O framework becomes more dominant relative to the vacancy-related features. However, upon decompression, these changes are largely reversible, with both the RE–O and V_O_ modes returning to their original positions and intensities, indicating elastic structural behavior and recovery of the initial local environment.

## 4 Conclusions

In conclusion, the synthesized (CePr)­O_2__–__δ_ and (CePrLa)­O_2__–__δ_ compounds demonstrate remarkable structural robustness under high pressure, retaining the fluorite-type cubic symmetry up to 30 GPa without undergoing any abrupt phase transitions. Both materials exhibit a distinctive compression anomaly in the 9–16 GPa range, observed as a plateau in their compressibility and a slope change in the RE–O Raman mode. This behavior is attributed to internal structural rearrangements, particularly distortions in bond angles, rather than a conventional crystallographic transition. In (CePrLa)­O_2−δ_, a reversible pressure-induced amorphization is observed above 22 GPa, as evidenced by the appearance and subsequent disappearance of a broad background signal in the low-angle diffraction region upon decompression. The bulk modulus values correlate with composition, with (CePr)­O_2__–__δ_ showing greater stiffness compared to (CePrLa)­O_2__–__δ_, while both materials exhibit a slight increase of compressibility beyond the anomaly. Raman spectroscopy further reveals the suppression of the F_2_g vibrational mode at ambient conditions due to cationic disorder, and its partial recovery under pressure, indicating improved local ordering upon compression. Meanwhile, the VO-related band remains structurally stable but evolves in intensity and shape, likely due to pressure-induced changes in Pr^3^^+^/Pr^4^^+^ redox states and vacancy redistribution. Upon decompression, both structural and vibrational features largely recover, suggesting the changes are elastic and reversible. Overall, these findings highlight the critical influence of configurational entropy and multicationic engineering in governing the high-pressure mechanical and vibrational behavior of rare-earth oxides, paving the way for their design and optimization in extreme environments.
